# Real-world treatment patterns, biomarker testing, and clinical outcomes of metastatic non-small cell lung cancer patients in the immunotherapy era

**DOI:** 10.3389/fonc.2024.1442909

**Published:** 2024-10-25

**Authors:** Lior Apter, Sarah Sharman Moser, Ashwini Arunachalam, Sivan Gazit, Moshe Hoshen, Gabriel Chodick, Nava Siegelmann-Danieli

**Affiliations:** ^1^ Health Division, Maccabi Healthcare Services, Tel Aviv, Israel; ^2^ Department of Health Policy and Management, School of Public Health, Faculty of Health Sciences, Ben-Gurion University of the Negev, Be’er-Sheva, Israel; ^3^ KSM Maccabi Institute for Research and Innovation, Maccabi Healthcare Services, Tel Aviv, Israel; ^4^ Outcomes Research, Value & Implementation, Merck & Co., Inc., Rahway, NJ, United States; ^5^ Faculty of Medicine, Tel Aviv University, Tel Aviv, Israel

**Keywords:** non-small cell lung cancer, tyrosine kinase inhibitors, survival, EGFR mutation, PD-L1 Inhibitors

## Abstract

**Background:**

Treatment for first-line (1L) metastatic non-small cell cancer (mNSCLC) changed with the introduction of immunotherapy. We describe treatment utilization and clinical outcomes in a real-world mNSCLC cohort in a 2.7-million-member state-mandated health provider.

**Methods:**

Newly diagnosed mNSCLC patients initiating systemic anti-cancer treatment (January 2017–December 2020) were identified from the National Cancer Registry. Real-world time on treatment (rwToT) was defined as the length of time between the first and last administration date of treatment. Real-world overall survival (rwOS) was estimated using Kaplan–Meier analysis. Outcomes were assessed at a minimum of 6 months’ follow-up (cutoff: 30 June 2021).

**Results:**

Among 843 patients, 85% had adenocarcinoma (NSQ) and 15% had squamous cell carcinoma (SQ) histology: of these, 43% and 26% were women, median age was 67 and 69 years, and 55% and 48% had 0–1 ECOG performance status, respectively (missing: 27% and 30%, respectively). Median follow-up for the entire cohort was 27.1 months (95% CI: 24.7–29.6). NSQ patients with no known EGFR/ALK/ROS1 aberrations received PD-1 inhibitor monotherapy (PDM) (*N* = 147) or combination (PDC) (*N* = 194) or platinum-based chemotherapy (PBC, *N* = 133). Median rwToT was 4.5 (95% CI: 3.5–7.6), 5.2 (95% CI: 4.6–7.6), and 2.3 (95% CI: 2.1–3.0) months, respectively; for the subgroup of patients with ECOG PS 0–1, rwToT was 9.4 (95% CI: 5.0–20.8), 7.1 (95% CI: 5.0–10.1), and 2.9 (95% CI: 2.2–4.1) months, respectively. Median rwOS from 1L was 12.5 (95% CI: 9.9–17.9), 14.8 (95% CI: 10.5–19.4), and 9.1 (95% CI: 7.1–11.5) months; for the subgroup of patients with ECOG PS 0–1, median rwOS was 25.1 [95% CI: 14.9–not reached (NR)], 17.6 (95% CI: 14.3–NR), and 11.3 (95% CI: 9.2–21.3) months, respectively. For ECOG PS 0–1 and PD-L1 ≥50% patients, median rwOS was 25.1 months (95% CI: 13.9–NR) and NR for PDM and PDC, respectively. For ECOG PS 0–1 and PD-L1 <50% patients, median rwOS was 14.3 (95% CI: 10.1–NR) and 11.2 (95% CI: 9.1–21.3) months for PDC and PBC, respectively.

**Conclusion:**

Our real-world data support the benefit of single-agent PD-1 inhibitor monotherapy for patients with PD-L1 high expression or PD-1 inhibitor combination for all patients diagnosed with mNSCLC with no known EGFR/ALK/ROS1 aberrations, initiating 1L treatment.

## 1 Introduction

Lung cancer is the second most common type of cancer diagnosed globally and the leading cause of cancer death worldwide (1–3). Non-small cell lung cancer (NSCLC) accounts for approximately 80%–90% of all lung cancers (2, 3) and includes squamous cell carcinoma, non-squamous carcinoma (adenocarcinomas, large cell, and undifferentiated carcinoma), and not otherwise specified (<5%) (4). Five-year survival for metastatic NSCLC (mNSCLC) patients was reported as only 6%–7% in a large database of patients diagnosed between 1999 and 2010, and an increase in survival has been seen from 2014 to 2018, due to several factors, including decrease in smoking and improved treatment options (2, 5, 6). Prior to introduction of immune checkpoint inhibitors (ICIs), median real-world overall survival (rwOS) for mNSCLC patients receiving platinum-based chemotherapy (PBC) has been poor (8.5 to 10 months), with a need for better and effective treatment options (7–9).

Targeted therapies and ICI, specifically programmed death-1/programmed death-ligand1 (PD-1/PD-L1) inhibitors, have become available over the last decade with the first ICI approved in 2015 for previously treated unresectable advanced/metastatic NSCLC (10). Over the years, pivotal clinical studies have demonstrated improved overall survival (OS) in patients treated with ICI versus PBC: In Keynote-407, median rwOS at 5 years follow-up for patients with squamous cell mNSCLC receiving pembrolizumab in combination with PBC was 17.2 (14.4 to 19.7) months vs. 11.6 (10.1 to 13.7) months with PBC alone (11); in Keynote-189, median rwOS at 5 years follow-up for patients with non-squamous mNSCLC was 22.0 (19.5 to 24.5) months with pembrolizumab in combination with PBC vs. 10.6 (8.7 to 13.6) months with PBC alone; in Keynote 024 trial, the median rwOS at 5 years follow-up for mNSCLC patients with programmed death-ligand 1 (PD-L1) tumor proportion score (TPS) ≥ 50% was 26.3 (18.3 to 40.4) months with pembrolizumab monotherapy vs. 13.4 (9.4 to 18.3) months with PBC alone (12, 13).

The guideline recommendations for systemic anti-cancer therapy for mNSCLC vary according to tumor histology and oncogenic actionable driver mutation status allowing for a personalized approach to treating the tumor (14, 15). National Comprehensive Cancer Network guidelines recommend that patients newly diagnosed with mNSCLC testing positive for actionable molecular biomarker or genomic tumor driver mutation, such as epidermal growth factor receptor (EGFR), anaplastic lymphoma kinase (ALK), or proto-oncogene tyrosine-protein kinase ROS enzyme (ROS1), be treated with a tyrosine kinase inhibitor (TKI) specific to the particular mutation or molecular biomarker-directed therapy (15). For patients with no known actionable genomic tumor driver mutations or molecular biomarkers, ICIs, specifically PD-1/PD-L1 inhibitor monotherapy or in combination with PBC (PD-1/PD-L1 inhibitor combination), are recommended.

The first PD-1/PD-L1 inhibitor single agent approved in Israel for mNSCLC was nivolumab monotherapy for second-line (2L) treatment and has been reimbursed in Israel since January 2016 (16). Based on the Keynote-024 trial, pembrolizumab has been reimbursed in Israel since January 2017, for first-line (1L) treatment as single-agent therapy for mNSCLC tumors with PD-L1 TPS ≥50% and no actionable mutations (EGFR/ALK) (17, 18). In January 2019, pembrolizumab in combination with chemotherapy was approved and reimbursed for 1L treatment of advanced NSCLC, regardless of PD-L1 expression level, based on Keynote-189 and Keynote-407 (11, 19–24). Atezolizumab monotherapy has been reimbursed in Israel since January 2020 for 2L treatment after PBC, and since January 2021 for 1L treatment of mNSCLC as monotherapy for patients with high PD-L1 expression (≥50%) and in combination with chemotherapy regardless of PD-L1 expression level based on the Impower110 study (25, 26).

Information from a real-world setting is important to understand the clinical effectiveness of PD-1/PD-L1 inhibitors in a wider population of patients beyond those considered eligible and included within clinical trials [Eastern Cooperative Oncology Group (ECOG) performance status (ECOG PS) 2 and beyond, comorbidities, etc.] (27). Since the approval of ICIs in 2016, cohort studies leveraging data from US community centers have demonstrated a median rwOS ranging from 16 to 21 months for 1L PD-1/PD-L1 inhibitor monotherapy and 15 to 19 months for 1L PD-1/PD-L1 inhibitor combination with PBC (28, 29). Similarly, a retrospective study conducted in the Netherlands reported a median rwOS of 15.8 months (95% CI: 9.4–22.1) for 1L PD-1/PD-L1 inhibitor monotherapy (30). A recent study conducted in Israel using electronic medical records from four Israeli cancer centers between the years 2016 and 2020 (*N* = 256, median follow-up of 22.3 months) have demonstrated a median rwOS of 12.5 (95% CI: 9.8–16.4) months and 20.4 [95% CI: 10.8–not reached (NR)] months in PD-1/PD-L1 inhibitor monotherapy and PD-1/PD-L1 inhibitor combination with chemotherapy group, respectively (31). This study had a relatively low number of patients (*N* = 256) and a significantly shorter follow-up for patients receiving PD-1/PD-L1 inhibitor combination therapy.

This real-world study builds on a previous study that reported evolving treatment patterns since the introduction of ICIs in Israel and reported high adherence to treatment guidelines (32). The objective of the present retrospective study was to further describe biomarker testing and treatment patterns by histology and evaluate OS for patients with no known actionable driver mutations and receiving 1L therapy since the approval of ICIs in Israel.

## 2 Methods

### 2.1 Data source

This retrospective cohort study was conducted on anonymized records derived from the computerized databases of Maccabi Healthcare Services (MHS), a nationwide healthcare insurer–provider. MHS has approximately 2.7 million members, representing over a quarter of the Israeli population and shares similar sociodemographic characteristics with the general population (33). The MHS database contains longitudinal data that are collected since 1993 for a stable population (with less than 1% of members moving out each year), including laboratory results from a single central laboratory, pharmacy prescription and purchase data, hospitalizations, procedures, and consultations. MHS uses the *International Classification of Diseases, Ninth Revision, Clinical Modification* (ICD-9-CM) coding systems, as well as self-developed coding systems to provide more granular diagnostic information. Procedures are coded using *Current Procedural Terminology* codes. MHS has developed several computerized registries of major chronic diseases, such as cardiovascular disease, hypertension, chronic obstructive pulmonary disease, chronic kidney disease, oncologic diseases, diabetes mellitus, and osteoporosis, to improve the quality of chronic care delivery to its members. The registries are continuously updated, and they identify patients via automatic search formulas, as opposed to being dependent solely upon active reporting by physicians (34–36).

In addition, data that were not available in the main database such as disease staging, imaging results [x-ray, computed tomography (CT), positron emission tomography-computed tomography (PET-CT), and magnetic resonance imaging (MRI)], histological type, metastases location, ECOG PS results, or genomic tumor driver mutation status were manually extracted from individual de-identified medical letters received from hospitalization discharge, day treatment in oncology units, and drug requests to the MHS Medication Approval Committee in MHS.

### 2.2 Study population

In this retrospective cohort study, we identified MHS members with a confirmed diagnosis of mNSCLC (from the MHS cancer registry or ICD-9-CM diagnosis codes in the MHS electronic database), between 1 January 2017 and 31 December 2020. The MHS cancer registry is compiled from pathology results from diagnosed cancer cases and from cancer treatment approvals by the MHS Medication Approval Committee.

Patients were included if they were at least 18 years of age at diagnosis, had at least 1 year of continuous healthcare enrollment in MHS before diagnosis date (to allow complete collection of baseline characteristics), and initiated systemic 1L treatment. Data were collected until 30 June 2021 to allow for at least 6 months of follow-up. Index date was set as the date of 1L treatment initiation.

### 2.3 Study variables

Demographic and clinical data collected included age at index, sex, socioeconomic status, district, prevalence of comorbid conditions, body mass index (BMI), and smoking status. Socioeconomic status was categorized into quartiles based on the poverty index of the member’s enumeration area at the neighborhood level (37, 38). The poverty index is based on several parameters including household income, educational level, crowding, physical conditions, and car ownership. BMI was defined as the closest to the index date within the 5-year period before the index date. Smoking data were collected from physician reporting and classified into ever, never, or unknown.

Comorbidities at baseline were identified using MHS registries (diabetes mellitus, cardiovascular disease, hypertension, osteoporosis, and chronic obstructive pulmonary disease) (34–36). The Deyo-Charlson comorbidity index (CCI), using ICD-9-CM codes and MHS registries to determine presence/absence of disease, was calculated (39). Imaging (x-ray, CT, PET-CT, and MRI) results, histology based on biopsy results (squamous cell, adenocarcinoma, and other), metastases location, genomic driver mutation testing, and PD-L1 testing were collected. PD-L1 testing results were categorized as PD-L1 TPS ≥50% or PD-L1 TPS <50% and type of assay was reported where recorded. Patients who tested negative for EGFR/ALK/ROS1 driver mutations or untested were defined as negative/unknown mutation status. ECOG PS was based on physician reporting in the medical records at baseline.

### 2.4 Treatment patterns

Treatment lines were defined according to the sequence of dispensed medications, with information captured both from the health maintenance organization (HMO) pharmacy database and from hospital medical records (including information on medications provided by private insurance and clinical studies). To capture combination regimens, medication(s) prescribed to the patient as written in medical letters and validated with purchase data within the first month (30 days) was considered to be within the same line of therapy. Addition of a new drug to a current regimen was considered a new treatment line, and cessation of a medication from a combination regimen (likely due to tolerance issues) was considered the same line.

Treatment patterns were described as changes from 1L, including moving to 2L and treatment discontinuation. Among patients who did not move to 2L during follow-up, discontinuation was defined as a treatment gap of >120 days from 1L date of administration or dispense + 1 day. Treatment gaps within each line were treatment interruption rather than discontinuation. Treatment patterns are presented by a class of anti-cancer therapy drugs that included PBC combination with or without vascular endothelial growth factor (VEGF) inhibitors, TKI therapy, PD-1/PD-L1 inhibitor monotherapy, and PD-1/PD-L1 inhibitor therapy in combination with PBC combination (PD-1/PD-L1 inhibitor combination).

### 2.5 Statistical analysis

Descriptive analyses were conducted to evaluate the demographic, clinical characteristics, and treatment patterns for the whole study cohort by histology (squamous cell carcinoma and adenocarcinoma). Categorical variables were reported as frequency and percentage, and continuous variables were reported as median [interquartile range (IQR)].

Outcomes were reported for a subcohort of patients with adenocarcinoma histology without actionable mutations. Time to event analysis for real-world time on treatment (rwToT) and rwOS was assessed using Kaplan–Meier analysis, and median time to event with 95% confidence intervals (CIs) is presented. For rwToT analysis, individuals were followed from the index date until the outcome (discontinuation of treatment), death, loss to follow-up, or end of follow-up period (30 June 2021), whichever occurred first. Patients were considered discontinued if they had a gap of 120 days or more since last dispensing or switched to 2L treatment.

Real-world OS was assessed using the all-cause mortality data from the National Insurance Institute. Individuals were followed from the index date until death, loss to follow-up, or end of study period (30 June 2021), whichever occurred first. All analyses are presented for 1L treatment. In a sub-analysis, rwToT and rwOS were assessed for patients with ECOG PS 0–1 and were further stratified by PD-L1 TPS <50% and PD-L1 TPS ≥50%.

All analyses were conducted using IBM SPSS Statistics for Windows, Version 22.0. Armonk, NY: IBM Corp and R version 3.5.1.

The study was approved by the local ethics review board of MHS in Israel.

## 3 Results

### 3.1 Whole study cohort—demographic, testing, and treatment patterns

This cohort consisted of 843 patients with histologically confirmed mNSCLC who initiated 1L treatment within the index period (1 January 2017 to 31 December 2020), with 85% (*n* = 714) adenocarcinoma and 15% (*n* = 1,295) squamous cell carcinoma. Data cutoff date was 30 June 2021. Median follow-up for the entire cohort was 27.1 (95% CI: 24.7–29.6) months.

For patients with adenocarcinoma, median age at the index date was 67 years (IQR 61–74), 43.1% were women, 72.7% were confirmed smokers, 22.1% had brain metastases, and 54.8% had ECOG PS 0–1. A total of 84.2% were tested for PD-L1, 92.2% tested for EGFR mutation, 82.5% tested for ALK translocation, and 71.3% tested for ROS1 translocation (**Table 1**). PD-L1 testing rates increased from 75.2% in 2017 to 85.6% in 2020, and most patients (76%) were tested within 30 days of mNSCLC diagnosis, with the majority of patients (93.2%) testing prior to 1L initiation (data not shown), and 35% of patients had a PD-L1 TPS ≥50% (**Table 1**). Most patients (63%) used the Dako 22C3 assay for PD-L1 test (others unknown).

**Table 1:** Baseline demographic characteristics of patients diagnosed with metastatic NSCLC by histology.

	Adenocarcinoma *n* = 714	Squamous cell carcinoma *n* = 129
Age at index, median [IQR]	67.00 [61.00, 74.00]	69.00 [64.00, 74.00]
18–34	3 (0.4)	
35–64	282 (39.5)	36 (27.9)
65–74	259 (36.3)	62 (48.1)
75+	170 (23.8)	31 (24.0)
Female sex, *n* (%)	308 (43.1)	34 (26.4)
District of residence, *n* (%)		
Center	446 (62.5)	84 (65.1)
North	138 (19.3)	26 (20.2)
South	130 (18.2)	19 (14.7)
Socioeconomic level, *n* (%)		
Low	266 (37.3)	50 (38.8)
Medium	157 (22.0)	23 (17.8)
High	291 (40.8)	56 (43.4)
Comorbidities		
Deyo Charlson comorbidity index^1^ mean (SD)	3.88 (3.35)	4.23 (3.36)
≤0	150 (21.0)	16 (12.4)
1–2	179 (25.1)	37 (28.7)
3–6	206 (28.9)	41 (31.8)
7+	179 (25.1)	35 (27.1)
Diabetes mellitus	173 (24.2)	49 (38.0)
Cardiovascular disease	211 (29.6)	53 (41.1)
Hypertension	358 (50.1)	71 (55.0)
Depression	149 (20.9)	28 (21.7)
Chronic obstructive pulmonary disease	112 (15.7)	49 (38.0)
Osteoporosis	183 (25.6)	33 (25.6)
Smoking ever—yes, *n* (%)	519 (72.7)	118 (91.5)
Body mass index, median [IQR]	25.76 [23.12, 29.42]	25.51 [22.33, 29.36]
ECOG performance status, *n* (%)		
0–1	391 (54.8)	62 (48.1)
2	88 (12.3)	19 (14.7)
3–4	43 (6.0)	9 (7.0)
Missing	192 (26.9)	39 (30.2)
Metastases, *n* (%)		
Brain	158 (22.1)	14 (10.9)
Lymph nodes	519 (72.7)	89 (69.0)
Liver	129 (18.1)	29 (22.5)
Adrenal glands	111 (15.5)	17 (13.2)
Bone	317 (44.4)	43 (33.3)
Tested for PD-L1 expression, *n* (%)	601 (84.2)	116 (89.9)
PD-L1 expression levels		
<50%	344 (48.2)	68 (52.7)
≥50%	252 (35.3)	48 37.2)
Missing	118 (16.5)	13 (10.1)
Genomic tumor driver mutation testing		
Tested for EGFR mutation, *n* (%)	658 (92.2)	109 (84.5)
Wild type	493 (69.0)	106 (82.2)
Mutant	163 (22.8)	3 (2.3)
Missing	58 (8.1)	20 (15.5)
Tested for ALK translocation, *n* (%)	589 (82.5)	45 (34.9)
Wild type	544 (76.2)	45 (34.9)
Mutant	45 (6.3)	
Missing	125 (17.5)	84 (65.1)
Tested for ROS1 translocation, *n* (%)	509 (71.3)	34 (26.4)
Wild type	493 (69.0)	34 (26.4)
Mutant	16 (2.2)	
Missing	205 (28.7)	95 (73.6)

^1^excluding HIV and malignancy.

ALK, anaplastic lymphoma kinase; BRAF, proto-oncogene B-Raf; ECOG, Eastern Cooperative Oncology Group; EGFR, epidermal growth factor receptor mutations; HIV, human immunodeficiency virus; IQR, interquartile range; NSCLC, non-small cell lung cancer; PD-L1, programmed death-ligand 1; ROS1, ROS proto-oncogene-1 receptor tyrosine kinase; TKI, tyrosine kinase inhibitors; T790M, Thr790Met.

For patients with squamous cell carcinoma, median age at index date was 69 years (IQR: 64–74), 26.4% were women, 91.5% were confirmed smokers, 10.9% had brain metastases, and 48.1% had ECOG PS 0–1 (**Table 1**). A total of 89.9% tested for PD-L1 expression, 84.5% tested for EGFR mutation, 34.9% tested for ALK translocation, and 26.4% tested for ROS1 translocation (**Table 1**). PD-L1 testing increased from 82.8% in 2017 to 96.6% in 2020, and most patients (67%) were tested within 30 days of mNSCLC diagnosis, with the majority of patients (90.2%) testing prior to 1L initiation (data not shown) and 37% of patients had a PD-L1 TPS ≥50% (**Table 1**). Overall, pembrolizumab was the most used PD-1/PD-L1 inhibitor therapy (97.7%) as monotherapy or in PD-1/PD-L1 inhibitor combination therapy (data not shown). For treatment patterns for 1L and 2L treatment irrespective of biomarker status, see **Supplementary Tables 2**, **3**.

### 3.2 Subcohort of patients with no actionable mutations

A total of 486 and 125 patients had adenocarcinoma or squamous cell carcinoma with no actionable driver mutations respectively (for demographic baseline data for patients with no actionable mutation, see **Supplementary Table 1**).

During the years 2017–2018, most patients with adenocarcinoma or squamous cell carcinoma with no actionable mutations and PD-L1 TPS ≥50% received PD-1/PD-L1 inhibitor monotherapy (96.8% and 91.3% respectively; **Tables 2A**, **B**). Since 2019, treatment was either PD-1/PD-L1 inhibitor monotherapy (48.5% and 50.0% respectively) or PD-1/PD-L1 inhibitor combination (49.5% and 37.5% respectively) for patients with PD-L1 TPS ≥50%. Among patients with adenocarcinoma or squamous cell carcinoma with no actionable mutations and PD-L1 TPS <50%, patients mostly received PBC combination from 2017 to 2018 (85.7% and 78.3%, respectively) and received PD-1/PD-L1 inhibitor combination from 2019 to 2020 (90.8% and 81.0%, respectively, **Tables 2A**, **B**).

**Table 2A:** First-line treatment patterns by PD-L1 testing status for adenocarcinoma mNSCLC patients without genomic tumor driver mutations, *n* = 486.

		Platinum-based chemotherapy *n* (%)	PD-1/PD-L1 inhibitor monotherapy	PD-1/PD-L1 inhibitor therapy with chemotherapy	Total*
**PD-L1 TPS<50**	**2017–2018**	90 (85.7)	4 (3.8)	5 (4.8)	105
	**2019–2020**	8 (5.7)	2 (1.4)	128 (90.8)	141
**PD-L1 TPS≥50**	**2017–2018**	1 (1.1)	90 (96.8)	2 (2.2)	93
	**2019–2020**	1 (1.0)	49 (48.5)	50 (49.5)	101
**Unknown**	**2017–2018**	26 (86.7)	1 (3.3)	1 (3.3)	30
	**2019–2020**	7 (43.8)	1 (6.3)	8 (50.0)	16
**Total**	**2017–2018**	117 (51.3)	95 (41.7)	8 (3.5)	228
	**2019–2020**	16 (6.2)	52 (20.2)	186 (72.1)	258

*Includes patients who received “other” treatment.

**Table 2B:** First-line treatment patterns by PD-L1 testing status for squamous cell mNSCLC patients without genomic tumor driver mutations, *n* = 125.

		Platinum-based chemotherapy *n* (%)	PD-1/PD-L1 inhibitor monotherapy	PD-1/PD-L1 inhibitor therapy with chemotherapy	Total*
**PD-L1 TPS<50**	**2017–2018**	18 (78.3)	2 (8.7)	1 (4.3)	23
	**2019–2020**	8 (19.0)	0 (0.0)	34 (81.0)	42
**PD-L1 TPS≥50**	**2017–2018**	1 (4.3)	21 (91.3)	1 (4.3)	23
	**2019–2020**	3 (12.5)	12 (50.0)	9 (37.5)	24
**Unknown**	**2017–2018**	3 (33.3)	4 (44.4)	0 (0.0)	9
	**2019–2020**	1 (25.0)	1 (25.0)	2 (50.0)	4
**Total**	**2017–2018**	22 (40.0)	27 (49.1)	2 (3.6)	55
	**2019–2020**	12 (17.1)	13 (18.6)	45 (64.3)	70

*Includes patients who received “other” treatment.

**Figure 1:**
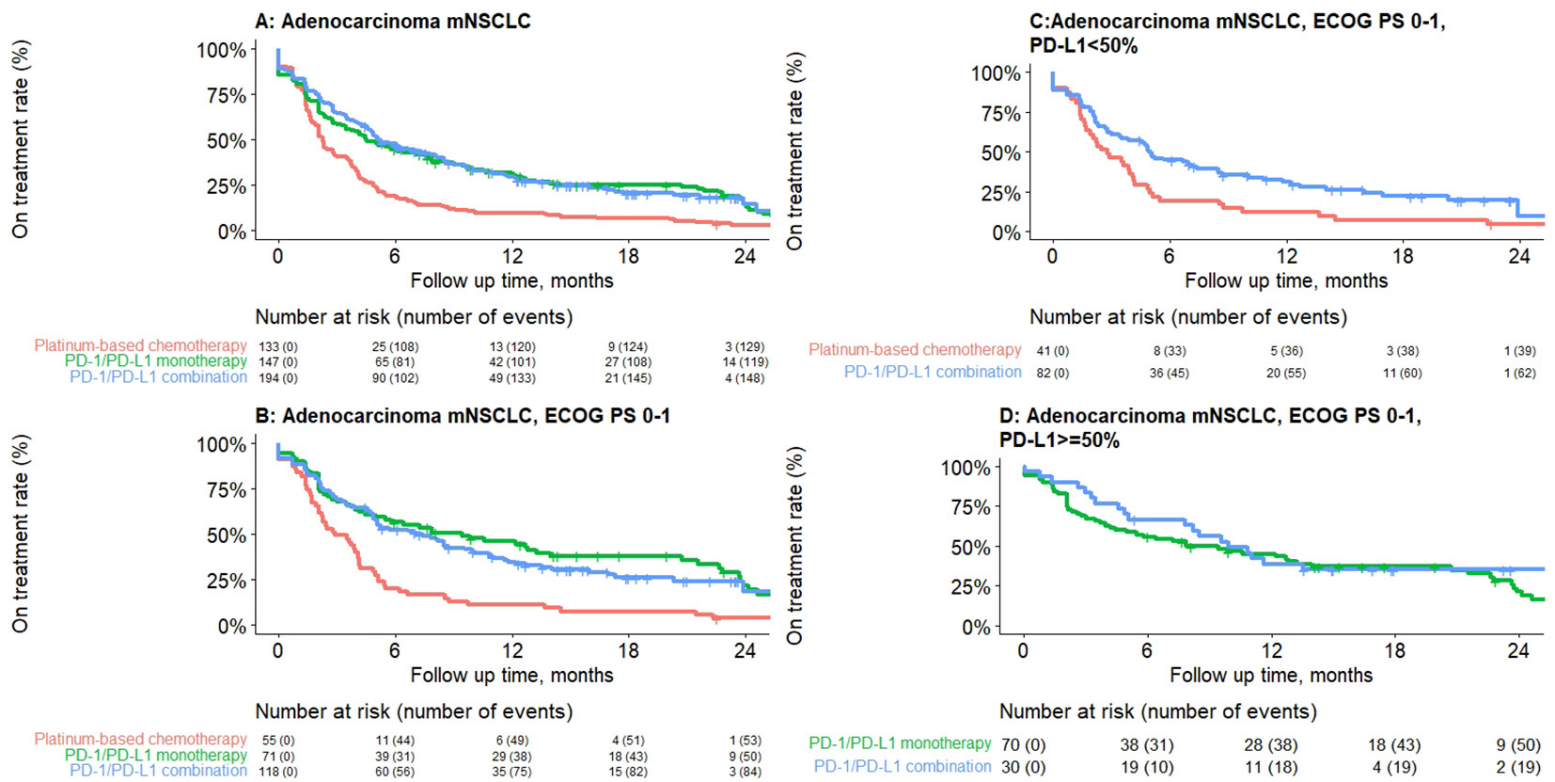
Kaplan–Meier plot depicting real-world time on treatment from first-line treatment initiation for adenocarcinoma mNSCLC patients without genomic tumor driver mutations, by treatment class. **(A)** Overall, **(B)** ECOG PS 0–1, **(C)** PD-L1 <50%, **(D)** PD-L1 ≥50%. Results presented are descriptive with no statistical comparisons.

For patients with adenocarcinoma:

Median rwToT was 4.5 (95% CI: 3.5–7.6), 5.2 (95% CI: 4.6–7.6), and 2.3 months (95% CI: 2.1–3.0) for PD-1/PD-L1 inhibitor monotherapy, PD-1/PD-L1 inhibitor combination, and PBC combination, respectively, for patients with overall ECOG PS (**Table 3**; **Figure 1**).

**Table 3:** Real-world time on treatment from first-line treatment initiation for adenocarcinoma mNSCLC patients without genomic tumor driver mutations, by treatment class.

	*N*	Number (%) discontinued	Median time on treatment (95% CI), mo	% on treatment rate at 6 mo	% on treatment rate at 12 mo	% on treatment rate at 24 mo
Adenocarcinoma, *n* = 486						
Platinum-based chemotherapy regimen	133	130 (97.7)	2.3 (2.1, 3.0)	18.8	9.8	2.8
PD-1/PD-L1 inhibitor monotherapy	147	131 (89.1)	4.5 (3.5, 7.6)	44.9	30.5	14.2
PD-1/PD-L1 inhibitor combination	194	149 (76.8)	5.2 (4.6, 7.6)	47.3	29.5	14.4
Adenocarcinoma and ECOG PS 0–1, *n* = 247						
Platinum-based chemotherapy regimen	55	54 (98.2)	2.9 (2.2, 4.1)	20.0	10.9	3.6
PD-1/PD-L1 inhibitor monotherapy	71	58 (81.7)	9.4 (5.0, 20.8)	56.3	45.8	21.6
PD-1/PD-L1 inhibitor combination	118	84 (71.2)	7.1 (5.0, 10.1)	52.4	34.6	18.0
Adenocarcinoma and ECOG PS 0–1 and PD-L1 <50%, *n* = 126						
Platinum-based chemotherapy regimen	41	40 (97.6)	2.8 (1.9, 4.2)	19.5	12.2	4.9
PD-1/PD-L1 inhibitor combination	82	62 (75.6)	5.0 (3.5, 8.5)	44.9	31.0	9.8
Adenocarcinoma and ECOG PS 0–1 and PD-L1 ≥50%, *n* = 101						
PD-1/PD-L1 inhibitor monotherapy	70	58 (82.9)	7.9 (4.4, 20.7)	55.7	45.0	21.2
PD-1/PD-L1 inhibitor combination	30	19 (63.3)	9.9 (7.8, NA)	66.7	38.6	35.1

Database cutoff date: 30 June 2021.

On treatment rates were based on the Kaplan–Meier estimates.

mo, months.

Results presented are descriptive with no statistical comparisons.

Excludes patients who received “other” treatment.

For a subgroup of patients with ECOG 0–1, rwToT was 9.4 (95% CI: 5.0–20.8), 7.1 (95% CI: 5.0–10.1), and 2.9 (95% CI: 2.2–4.1) months for patients who received PD-1/PD-L1 inhibitor monotherapy, PD-1/PD-L1 inhibitor combination, and PBC combination, respectively. For patients with ECOG PS 0–1 and PD-L1 TPS ≥50%, rwToT was 7.9 (95% CI: 4.4–20.7) and 9.9 months (95% CI: 7.8–NR) for PD-1/PD-L1 inhibitor monotherapy and PD-1/PD-L1 inhibitor combination, respectively. For patients with ECOG PS 0–1 and PD-L1 TPS <50%, rwToT was 5.0 (95% CI: 3.5–8.5) and 2.8 months (95% CI: 1.9–4.2) for PD-1/PD-L1 inhibitor combination and PBC, respectively (**Table 3**; **Figure 1**).

Median rwOS was 11.3 months (95% CI: 9.9–13.4) for the overall adenocarcinoma cohort with no actionable mutations, and 12.5 (95% CI: 9.9–17.9), 14.8 (95% CI: 10.5–19.4), and 9.1 (95% CI: 7.1–11.5) months for patients who received PD-1/PD-L1 inhibitor monotherapy, PD-1/PD-L1 inhibitor combination, and PBC combination, respectively (**Table 4**). Furthermore, for the subgroup of patients with ECOG PS 0–1, median rwOS was 25.1 (95% CI: 14.9–NR), 17.6 (95% CI: 14.3–NR), and 11.3 (95% CI: 9.2–21.3) months for those who received PD-1/PD-L1 inhibitor monotherapy, PD-1/PD-L1 inhibitor combination, and PBC combination respectively (**Table 4**).

**Table 4:** Overall survival from first-line treatment initiation for adenocarcinoma mNSCLC patients without genomic tumor driver mutations, by treatment class.

	*N*	Number died. *n* (%)	Median real-world overall survival (95%, CI), mo	% survival rate at 6 mo	% survival rate at 12 mo	% survival rate at 24 mo
Adenocarcinoma, *n* = 486						
Platinum-based chemotherapy regimen	133	109 (82.0)	9.1 (7.1, 11.5)	63.9	39.8	29.1
PD-1/PD-L1 inhibitor monotherapy	147	96 (65.3)	12.5 (9.9, 17.9)	68.0	52.2	36.9
PD-1/PD-L1 inhibitor combination	194	109 (56.2)	14.8 (10.5, 19.4)	71.1	54.3	38.7
Adenocarcinoma and ECOG PS 0–1, *n* = 247						
Platinum-based chemotherapy regimen	55	41 (74.5)	11.3 (9.2, 21.3)	76.4	47.3	36.4
PD-1/PD-L1 inhibitor monotherapy	71	38 (53.5)	25.1 (14.9, NR)	80.3	62.5	50.7
PD-1/PD-L1 inhibitor combination	118	56 (47.5)	17.6 (14.3, NR)	79.7	62.3	46.0
Adenocarcinoma and ECOG PS 0–1 and PD-L1 <50, *n* = 126						
Platinum-based chemotherapy regimen	41	30 (73.2)	11.2 (9.1, 21.3)	75.6	46.3	34.1
PD-1/PD-L1 inhibitor combination	82	44 (53.7)	14.3 (10.1, NR)	75.6	55.2	39.0
Adenocarcinoma and ECOG PS 0–1 and PD-L1 ≥50, *n* = 101						
PD-1/PD-L1 inhibitor monotherapy	70	38 (54.3)	25.1 (13.9, NR)	80.0	61.9	50.3
PD-1/PD-L1 inhibitor combination	30	11 (36. 7)	NR	86.7	76.5	58.2

Database cutoff date: 30 June 2021.

On treatment rates were based on the Kaplan–Meier estimates.

mo, months.

Results presented are descriptive with no statistical comparisons.

Excludes patients who received “other” treatment.

**Figure 2:**
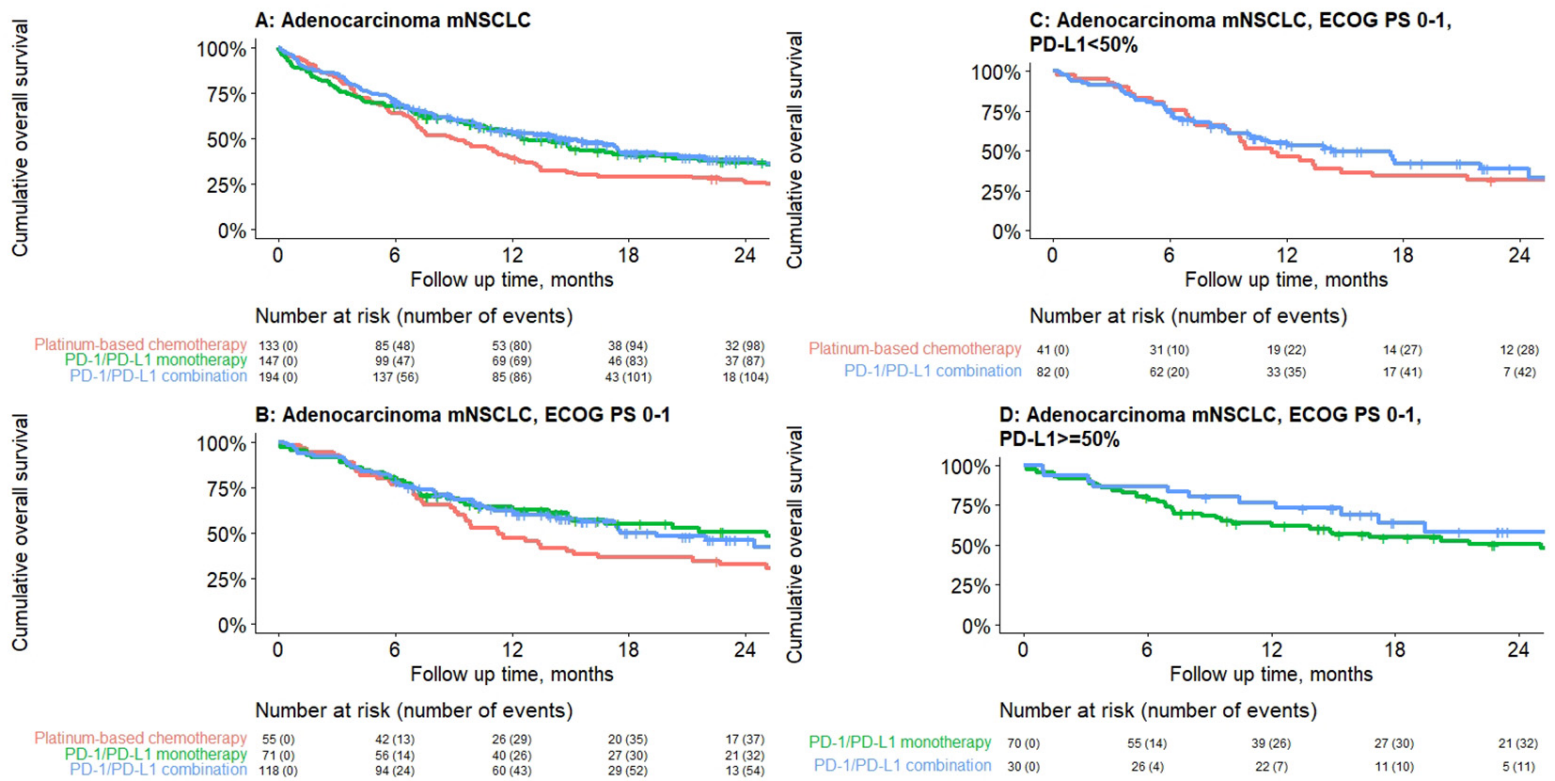
Median real-world overall survival by treatment class for adenocarcinoma mNSCLC patients without genomic tumor driver mutations initiating first-line treatment, by treatment class. **(A)** Overall, **(B)** ECOG PS 0–1, **(C)** PD-L1 <50%, **(D)** PD-L1 ≥50%. Results presented are descriptive with no statistical comparisons.

For patients with ECOG PS 0–1 and PD-L1 TPS ≥50%, median rwOS was 25.1 months (95% CI: 13.9–NR) and NR for PD-1/PD-L1 inhibitor monotherapy and PD-1/PD-L1 inhibitor combination, respectively (**Table 4**). At 24 months, 50.3% and 58.2% of patients who received PD-1/PD-L1 inhibitor monotherapy and PD-1/PD-L1 inhibitor combination, respectively, were still alive. For patients with ECOG PS 0–1 and PD-L1 TPS <50%, median rwOS was 14.3 (95% CI: 10.1–NR) and 11.2 (95% CI: 9.1–21.3) months for PD-1/PD-L1 inhibitor combination and PBC, respectively (**Table 4**; **Figure 2**).

A total of 190 (39.1%) patients with adenocarcinoma without actionable mutations continued to receive 2L treatment (data not shown). Clinical outcomes for squamous cell carcinoma patients with no actionable mutation are not presented due to the small sample size (<50).

## 4 Discussion

This retrospective study described baseline characteristics, biomarker testing patterns, treatment patterns, and outcomes for a real-world cohort of unselected patients diagnosed with mNSCLC between 2017 and 2020 after availability of ICIs in a large Israeli healthcare service.

Majority of the patients in this cohort had adenocarcinoma histology (85%), similar to other recent real-world studies (28, 30, 31, 40–42). The squamous cell cohort patients were slightly older, had a higher comorbidity index and lower performance status, and were more likely to be confirmed smokers compared to patients with adenocarcinoma. The median age and smoking status in the overall cohort were similar to other studies (9, 28, 30, 31, 43–47).

EGFR mutations, ALK, and ROS1 translocations were identified almost exclusively in patients with adenocarcinoma in line with reported scientific literature (48, 49). PD-L1 testing was approved and reimbursed in the Israel National Formulary in January 2017, and we found high rates of testing, increasing from 2017 to 2020, with the majority of testing carried out within 30 days of diagnosis and before the initiation of 1L treatment in line with testing and treatment guidelines (50). We found a third of the patients with PD-L1 TPS ≥50%, which is consistent with and similar to published literature (32, 41, 51).

1L treatment patterns were consistent with national treatment guidelines and the list of reimbursed drugs included in the Israel national formulary. Patients with an actionable molecular biomarker or genomic tumor driver mutation mostly received TKIs, and patients with no actionable mutations and with PD-L1 TPS ≥50% mostly received PD-1/PD-L1 inhibitor monotherapy through the years. The utilization of PD-1/PD-L1 inhibitor combination was mostly among patients with PD-L1 TPS<50% and increased with its inclusion in the Israel National Formulary since January 2019. The decision whether to use PD-1/PD-L1 inhibitor monotherapy or PD-1/PD-L1 inhibitor combination for patients with PD-L1 TPS ≥50% from January 2019 was based on the discretion of the treating physician. Pembrolizumab was the most used ICI as monotherapy or in combination with PBC (52).

The findings are similar to a recently published paper that shows that from 2016 to 2020, the 1L treatment approach for advanced NSCLC in the US evolved from anti-PD-1/PD-L1 monotherapy to combination chemo-immunotherapy, alongside a rise in biomarker testing (47).

However, a recent real-world study conducted in five European countries, which included patients diagnosed with mNSCLC during the COVID-19 pandemic in 2020, showed that chemotherapy usage remained widespread despite guidelines recommending immunotherapy-based 1L treatment for mNSCLC. That study also highlighted the significant impact of the COVID-19 pandemic on patient management (51).

RwToT serves as a proxy for progression-free survival (PFS) in the real-world setting, with the assumption that patients continued their 1L treatment if they had clinical benefit and switched or discontinued treatment upon disease progression or toxicity or death. This measure has been published in previous real-world studies (53, 54). Median rwToT for patients receiving PD-1/PD-L1 inhibitor monotherapy and for PD-1/PD-L1 inhibitor combination in our study was consistent with published real-world studies (46, 55–59).

Our study findings in the subset of patients with ECOG PS 0–1 were also consistent with the analogous endpoint of PFS in KN024 [mNSCLC, median PFS pembrolizumab arm, 7.7 (6.1–10.2) months]; KN042 [mNSCLC with PD-L1 TPS ≥ 50%, median PFS pembrolizumab arm, 6.5 (5.9–8.8) months]; and KN189 [non-squamous mNSCLC, median PFS pembrolizumab arm, 7.5 (5.1–10.5) months] (13, 60, 61).

Until recently, the standard of care for patients with mNSCLC with no actionable mutations was PBC, with or without VEGF inhibitors. Real-world data from a large US community practice in the pre-immunotherapy era reported a median OS of 10 months for adenocarcinoma and 8.5 months for squamous cell carcinoma, similar to our cohort who received PBC (9). In the overall patient population, median rwOS for patients with ECOG PS 0–1 was comparable with clinical trial data that found 69.8%/45.7% and 48.0%/27.3% survival at 12/24 months for PD-1/PD-L1 inhibitor-based regimens and PBC, respectively, in the KN189 trial (61) and 70.3%/51.5% survival at 12/24 months for PD-1/PD-L1 inhibitor monotherapy in the KN024 trial (62) with similar results in the IMPOWER110 trial (63). The results are also comparable with a recently published real-world observational study from central Eastern Europe suggesting the similar effectiveness of 1L PD-1/PD-L1 with or without chemotherapy in patients with advanced NSCLC to those observed in randomized clinical trials (42).

Real-world practice includes patients with all performance status eligible for treatment, and not only ECOG PS 0–1 as in clinical trials. An observational study conducted in Denmark reported a median rwOS of 15 (12–17) months for non-squamous patients who received PD-1/PD-L1 inhibitor combination with variable ECOG status, similar to our results (28).

The findings from a study in central Switzerland also indicate that treatment with checkpoint inhibitors enhances OS in patients with mNSCLC and that PD-L1 expression may serve as a predictive marker in patients treated outside of clinical trials (41).

A third of the patients in this cohort had PD-L1 TPS ≥50%, for whom treatment options include either PD-1/PD-L1 inhibitor monotherapy or PD-1/PD-L1 inhibitor chemotherapy combinations. To our knowledge, there are no randomized clinical trials reporting comparative data on monotherapy vs. combination (28, 29, 31, 42, 52, 64). For patients who received 1L PD-1/PD-L1 inhibitor monotherapy, we found a median rwOS of 12.5 months for patients with variable performance status, and 25.1 months for those with ECOG PS 0–1 (50.3% of patients were still alive at 24 months). For patients receiving PD-1/PD-L1 inhibitor chemotherapy combination, the median rwOS was 14.8 months for patients with variable performance status and was not reached (58.2% were still alive at 24 months) for those with ECOG PS 0–1. Our results are in line with several other real-world studies including the United States, the Netherlands, Central Europe, and Israel (29–31, 46, 57, 58, 65–67). It is important to note that in some of these studies, the percentage of ECOG PS 0–1 patients was higher than in our study.

Our study results show that for mNSCLC patients with no actionable mutations and PD-L1 TPS ≥50% and initiating 1L systemic therapy in the real world, both PD-1/PD-L1 inhibitor monotherapy and PD-1/PD-L1 inhibitor combination are effective treatment options. Patient and clinical characteristics including physician or patient preference may be considered when choosing a treatment option.

The strengths of this study include high-quality longitudinal data obtained from the MHS electronic database including all medical and billing data, and a comprehensive review of patient medical records with a long follow-up. MHS comprises 25% of the patient population and shows real-world generalizability within the population (33). Our study reflects current practice patterns and is the first study to report therapy utilization and outcomes based on patients with no known actionable driver mutations in an Israeli real-world cohort. However, this study included only those patients who initiated 1L therapy and does not provide insight into untreated patients.

**Supplementary Tables 4A**, **B** summarize real-world published data of mNSCLC patients including patient characteristics and outcomes.

Limitations of our findings include the retrospective nature of this study. We found that approximately a third of patients had missing ECOG PS; these patients had slightly lower ToT and OS as compared to patients with ECOG PS 0–1 (data not shown), and we hypothesize that this was because of the lack of documentation in the medical notes by physicians. Race was not available in our dataset; however, based on the demographics of Israel, we know that most patients were Caucasian. Actionable driver mutation information was only available for those who were tested. Owing to the small sample size of certain subgroups, including patients (ECOG PS 0–1) with PD-L1 ≥50% receiving PD-1/PD-L1 inhibitor combination, results should be interpreted with caution. Further research should explore the evolution of treatment patterns and associated outcomes following the changing treatment landscape, including long-term outcomes for patients receiving ICIs.

## 5 Conclusion

We describe adoption of biomarker testing and initiation of guideline concordant treatment in Israel after introduction of ICIs. Our real-world data demonstrate the real-world effectiveness of a single-agent PD-1/PD-L1 inhibitor or PD-1/PD-L1 inhibitor combination treatment in an upfront setting for patients with PD-L1 overexpressing tumors and PD-1/PD-L1 inhibitor combination for patients irrespective of PD-L1 expression level.

## Data Availability

The datasets generated and/or analyzed during the current study are not publicly available due the Israeli Ministry of Health and MHS’s data privacy policy but are available from the corresponding author on reasonable request. Requests to access the datasets should be directed to moser_sa@mac.org.il.
