# Supplementary material for: Real-world treatment patterns, biomarker testing, and clinical outcomes of metastatic non-small cell lung cancer patients in the immunotherapy era
Source: Front Oncol. 2024 Oct 25;14:1442909. doi: 10.3389/fonc.2024.1442909 (PMC11543355; doi:10.3389/fonc.2024.1442909)
Supplement: Supplementary file 1 [file Table1.docx]

**Supplement: Real-world treatment patterns, biomarker testing and clinical outcomes of metastatic non-small cell lung cancer patients in the immunotherapy era.**

**Supplementary Table 1:** Baseline demographic characteristics of patients diagnosed with mNSCLC without genomic tumor actionable mutations, n=611.

|  | **Adenocarcinoma** | | | | **Squamous Cell Carcinoma, n=125** |
| --- | --- | --- | --- | --- | --- |
|  | **Overall, n=486** | **Platinum based chemotherapy regimen, n=133** | **PD-1/PD-L1 inhibitor monotherapy, n=147** | **PD-1/PD-L1 inhibitor therapy with chemotherapy,**  **n=194** |  |
| **Age at index, median [IQR]** | **68.00 [61.00, 74.00]** | **67.00 [62.00, 75.00]** | **68.00 [62.00, 75.00]** | **67.00 [61.00, 72.00]** | **69.00 [64.00, 74.00]** |
| 18 - 34 | 1 (0.2) | 0 ( 0.0) | 0 (0.0) | 1 (0.5) | 0 |
| 35 - 64 | 190 (39.1) | 54 (40.6) | 51 (34.7) | 79 (40.7) | 34 (27.2) |
| 65 - 74 | 181 (37.2) | 43 (32.3) | 57 (38.8) | 80 (41.2) | 61 (48.8) |
| 75+ | 114 (23.5) | 36 (27.1) | 39 (26.5) | 34 (17.5) | 30 (24.0) |
| **Female sex, n (%)** | **174 (35.8)** | **45 (33.8)** | **53 (36.1)** | **71 (36.6)** | **33 (26.4)** |
| **District** |  |  |  |  |  |
| Centre | 302 (62.1) | 84 (63.2) | 98 (66.7) | 112 (57.7) | 81 (64.8) |
| North | 102 (21.0) | 24 (18.0) | 30 (20.4) | 46 (23.7) | 25 (20.0) |
| South | 82 (16.9) | 25 (18.8) | 19 (12.9) | 36 (18.6) | 19 (15.2) |
| **Socioeconomic status, n (%)** |  |  |  |  |  |
| Low | 187 (38.5) | 63 (47.4) | 50 (34.0) | 68 (35.1) | 48 (38.4) |
| Medium | 106 (21.8) | 26 (19.5) | 39 (26.5) | 40 (20.6) | 22 (17.6) |
| High | 193 (39.7) | 44 (33.1) | 58 (39.5) | 86 (44.3) | 55 (44.0) |
| **Co-morbidities** |  |  |  |  |  |
| **Deyo Charlson co-morbidity index^1^, mean (SD)** | **3.99 (3.37)** | **3.68 (3.42)** | **3.93 (3.30)** | **4.13 (3.34)** | **4.27 (3.39)** |
| <= 0 | 98 (20.2) | 28 (21.1) | 27 (18.4) | 42 (21.6) | 16 (12.8) |
| 1 - 2 | 122 (25.1) | 40 (30.1) | 42 (28.6) | 38 (19.6) | 35 (28.0) |
| 3 - 6 | 133 (27.4) | 33 (24.8) | 39 (26.5) | 57 (29.4) | 39 (31.2) |
| 7+ | 133 (27.4) | 32 (24.1) | 39 (26.5) | 57 (29.4) | 35 (28.0) |
| Diabetes mellitus | 121 (24.9) | 32 (24.1) | 36 (24.5) | 51 (26.3) | 46 (36.8) |
| Cardiovascular disease | 165 (34.0) | 48 (36.1) | 55 (37.4) | 58 (29.9) | 50 (40.0) |
| Hypertension | 90 (18.5) | 30 (22.6) | 28 (19.0) | 31 (16.0) | 68 (54.4) |
| Depression | 132 (27.2) | 38 (28.6) | 44 (29.9) | 49 (25.3) | 26 (20.8) |
| Chronic obstructive pulmonary disease | 28 (5.8) | 12 (9.0) | 9 (6.1) | 6 (3.1) | 47 (37.6) |
| Osteoporosis | 115 (23.7) | 31 (23.3) | 40 (27.2) | 42 (21.6) | 33 (26.4) |
| **Smoking, never or unknown, n (%)** | **62 (12.8)** | **27 (20.3)** | **18 (12.2)** | **16 (8.2)** | **10 (8.0)** |
| **Body mass index, median [IQR]** | **25.35 [22.49, 29.33]** | **25.21 [23.11, 30.22]** | **24.77 [21.92, 28.73]** | **26.13 [23.47, 29.03]** | **25.39 [22.26, 28.86]** |
| **ECOG performance status, n (%)** |  |  |  |  |  |
| 0-1 | 247 (50.8) | 55 (41.4) | 71 (48.3) | 118 (60.8) | 59 (47.2) |
| 2 | 68 (14.0) | 20 (15.0) | 18 (12.2) | 30 (15.5) | 19 (15.2) |
| 3-4 | 32 (6.6) | 3 (2.3) | 14 (9.5) | 14 (7.2) | 8 (6.4) |
| missing | 139 (28.6) | 55 (41.4) | 44 (29.9) | 32 (16.5) | 39 (31.2) |
| **Metastases, n (%)** |  |  |  |  |  |
| Brain | 91 (18.7) | 15 (11.3) | 28 (19.0) | 46 (23.7) | 13 (10.4) |
| Lymph nodes | 348 (71.6) | 84 (63.2) | 99 (67.3) | 156 (80.4) | 86 (68.8) |
| Liver | 82 (16.9) | 18 (13.5) | 25 (17.0) | 39 (20.1) | 29 (23.2) |
| Adrenal glands | 85 (17.5) | 27 (20.3) | 30 (20.4) | 24 (12.4) | 17 (13.6) |
| Bone | 204 (42.0) | 60 (45.1) | 55 (37.4) | 82 (42.3) | 42 (33.6) |
| **Tested for PDL1 expression, n (%)** | 443 (91.2) | 101 (75.9) | 145 (98.6) | 187 (96.4) | 112 (89.6) |
| **PD-L1 expression levels** |  |  |  |  |  |
| <50% | 246 (50.6) | 98 (73.7) | 6 (4.1) | 133 (68.6) | 65 (52.0) |
| >=50 % | 194 (39.9) | 2 (1.5) | 139 (94.6) | 52 (26.8) | 47 (37.6) |
| Missing | 46 (9.5) | 33 (24.8) | 2 (1.4) | 9 (4.6) | 13 (10.4) |
| 1- excluding HIV and malignancy.  ECOG, Eastern Cooperative Oncology Group; HIV, human immunodeficiency virus; IQR, inter-quartile range; NSCLC, non-small cell lung cancer; PD-L1, programmed death-ligand 1; TKI, tyrosine kinase inhibitors |  |  |  |  |  |

**Supplementary Table 2:** First-line treatment patterns for patients with adenocarcinoma and squamous cell carcinoma mNSCLC, n=843

|  |  | Platinum-based chemotherapy  n (%) | TKI inhibitor therapy  n (%) | PD-1/PD-L1 inhibitor monotherapy  n (%) | PD-1/PD-L1 inhibitor combination  n (%) |
| --- | --- | --- | --- | --- | --- |
| **Adenocarcinoma** | **2017-2018** | 119 (35.8) | 97 (29.2) | 97 (29.2) | 9 (2.7) |
|  | **2019-2020** | 16 (4.2) | 118 (30.9) | 53 (13.9) | 191 (50.0) |
| **Squamous cell**  **carcinoma** | **2017-2018** | 22 (40.0) | 1 (1.8) | 27 (49.1) | 2 (3.6) |
|  | **2019-2020** | 12 (16.2) | 2 (2.7) | 13 (17.6) | 47 (63.5) |
| Excludes ‘other’ treatment  Includes all patients irrespective of any biomarker status | | | | | |

**Supplementary Table 3:** First and second-line treatment patterns by histology for patients with adenocarcinoma and squamous cell carcinoma mNSCLC.

| **Treatment class** | **1L**  **n (%)** | **2L**  **n (%)** |
| --- | --- | --- |
| **Adenocarcinoma** | **N=714** | **N=284** |
| Platinum-based chemotherapy | 135 (18.9) | 58 (20.5) |
| TKI inhibitor therapy | 215 (30.1) | 77 (27.1) |
| PD-1/PD-L1 inhibitor monotherapy | 150 (21.0) | 73 (25.7) |
| PD-1/PD-L1 inhibitor therapy with chemotherapy | 200 (28.0) | 26 (9.2) |
| Other | 14 (2.0) | 50 (17.6) |
| **Squamous cell carcinoma** | **N=129** | **N=54** |
| Platinum-based chemotherapy | 34 (26.4) | 10 (18.5) |
| TKI inhibitor therapy | 3 (2.3) | 0 (0.0) |
| PD-1/PD-L1 inhibitor monotherapy | 40 (31.0) | 24 (44.4) |
| PD-1/PD-L1 inhibitor therapy with chemotherapy | 49 (38.0) | 4 (7.4) |
| Other | 3 (2.3) | 16 (29.6) |
| PD-L1, programmed death-ligand 1  Includes all patients irrespective of biomarker status | | |

**Supplementary Table 4a:** Summary of real-world data patient characteristics.

| **Author** | **Reference Number** | **Year** | **N** | **Countries included** | **Racial distribution of patients** | **Age (years)** | **Male (%)** | **Current or Former Smoking (%)** | **Adenocarcinoma/ Non-Squamous (%)** | **ECOG PS 0-1 (%)** | **PD-L1 TPS >=50 (%)** |
| --- | --- | --- | --- | --- | --- | --- | --- | --- | --- | --- | --- |
| Abernethy AP | 9 | 2017 | 2014 | United States | 62.6% Caucasian, 8.3% Black, 1.8% Asian, 27.3% Unknown | 67.4 (mean) | 55.1 | 86.7 | 78.7 |  |  |
| Izano MA | 28 | 2023 | 739 | Netherlands, Denmark | 82.1% Caucasian, 12.3% Black, 1.5% Asian, 4.1% Unknown | 67 (median) | 52.2 | 67.5 | 79.2 | 15.8 | 33.7 |
| Kehl KL | 29 | 2021 | 19529 | United States | 89% White, 7% Black, 1% Hispanice, 4% Other | 73.8 (median) | 53.8 |  |  |  |  |
| Cramer-van der Welle CM | 30 | 2021 | 1950 | Netherlands, Denmark |  | 66 (Median) | 54 |  | 87 | 96 | 100 |
| Dudnik E | 31 | 2021 | 256 | Israel |  | 67 (Mean) | 66.4 | 90.6 | 77 | 71.1 |  |
| Ivanović M | 40 | 2021 | 66 | Slovenia |  | 64 (Median) | 55 | 82 | 83 | 94 | 55 |
| Allmann V | 41 | 2023 | 274 | Switzerland |  | 68.4 (Median) | 61.7 | 65 | 73 |  | 20.8 |
| Pelicon V | 42 | 2023 | 176 | Slovenia |  | 64 (Median) | 60.8 | 88.6 | 79.5 | 84.1 | 69.9 |
| Morinaga D | 46 | 2023 | 676 | Japan |  | 67 (Median) | 72.5 | 84.9 | 61.4 | 81 | 21.6 |
| Divan HA | 47 | 2024 | 2175 | United States | 76.8% White, 13.2% Black, 2.4% Asian, 7.5% Other | 68 (Median) | 53.7 | 88 | 66.5 | 64.7 | 5.3 |
| Bailey H | 51 | 2023 | 1073 | France, Germany, Italy, Spain, United Kingdom |  | 66.2 (Mean) | 65.1 |  | 63.7 | 81.7 | 36 |
| Velcheti V | 55 | 2019 | 524 | United States | 66.6% White, 9.7% Black, 3.1% Asian, 9.9% Other, 10.7% Unknown | 72 (Median) | 52.3 | 92.9 | 70.6 | 73.7 | 100 |
| Velcheti V | 56 | 2022 | 1044 | United States | 69.4% White, 8.7% Black, 2.0% Asian, 9.2% Other, 10.6% Unknown | 73 (Median) | 50.6 | 93.2 | 68.4 | 77.3 | 100 |
| Nokihara H | 57 | 2022 | 1208 | Japan |  | 70 (Median) | 80.7 | 90.1 | 61.6 | 53.6 | 43.4 |
| Velcheti V | 58 | 2021 | 238 | United States | 74.0% White, 12.8% Black, 2.0% Asian, 0.4% Hispanic or Latino, 10.8% other | 66 (Median) | 59.4 | 91.9 | 100 | 100 | 27.9 |
| Waterhouse D | 59 | 2022 | 11445 | United States |  |  |  |  |  |  |  |
| Pérol M | 64 | 2022 | 520 | United States | 69.8% White, 8.1% Black, 11.2%Other, 11.0% Missing | 70 (Median) | 50.4 | 90.4 | 100 | 100 | 100 |
| Velcheti V | 66 | 2022 | 566 | United States | 75.8% White, 9.7% Black, 3.4% Asian, 11.1% Other | 71 (Median) | 52.7 | 92.6 | 71.6 | 100 | 100 |
| Leonetti A | 67 | 2024 | 1068 | Italy |  | 66 (Median) | 63.8 | 82.4 |  | 90.5 | 3.6 |

**Supplementary Table 4b:** Summary of real-world data patient outcomes.

| **Author** | **Reference Number** | **PD-1/PD-L1 inhibitor monotherapy mOS, months (95%CI)** | **PD-1/PD-L1 inhibitor combination mOS, months (95%CI)** | **PD-1/PD-L1 inhibitor monotherapy mPFS, months (95%CI) (** | **PD-1/PD-L1 inhibitor combination mPFS, months (95%CI)** |
| --- | --- | --- | --- | --- | --- |
| Abernethy AP | 9 |  |  |  |  |
| Izano MA | 28 | 18 (14-22) | 13 (11-15) |  |  |
| Kehl KL | 29 | 11.4 (10.5-12.3) | 12.9 (11.8-14.0) |  |  |
| Cramer-van der Welle CM | 30 | 15.8 (9.4–22.1) |  | 8.9 (3.7–14.1) |  |
| Dudnik E | 31 | 12.5 (9.8-16.4) | 20.4 (10.8-NR) | 4.9 (3.1-7.6) time to treatment discontinuation | 8.0 (4.7-115.6) time to treatment discontinuation |
| Ivanović M | 40 | NR (7.1-NR) for 1L cohort, N=26 |  | 9.3 (3.5-NR) for 1L cohort, N=26 |  |
| Allmann V | 41 | 15 (12-20) |  |  |  |
| Pelicon V | 42 | 19.4 (11.1-27.6) | 21.3 (15.9-26.7) |  |  |
| Morinaga D | 46 | 12.3 (9.9-18.2) - 12.7 (11.6-14.3) |  | 3.3 (2.8-3.7) - 4.8 (3.5-6.1) |  |
| Divan HA | 47 |  |  |  |  |
| Bailey H | 51 |  |  |  |  |
| Velcheti V | 55 |  |  | 6.9 (5.6-8.3) for PS 0-1 and time on treatment |  |
| Velcheti V | 56 |  |  | 7.4 (6.3-8.1) for PS 0-1 for time on treatment and PS-L1 >=50% |  |
| Nokihara H | 57 | NR |  | 9.7 (8.1–11.1) |  |
| Velcheti V | 58 | 16.5 (13.2-20.6) |  | 6.4 (5.4-7.8) |  |
| Waterhouse D | 59 | 13.1 | 11.7 | 4.7 | 5.6 |
| Pérol M | 64 | 22.1 (18.3-30.3) | 21.0 (15.3-NR)) | 11.5 (8.1-15.0) | 10.8 (9.0-15.3) |
| Velcheti V | 66 | 19.6 (16.6-24.3) |  |  |  |
| Leonetti A | 67 |  | 16.1 (14.4-18.8) |  | 9.9 (8.8-11.2) |
